# Analysis of Calcaneal Fracture-Related Complications—A Retrospective Chart Review

**DOI:** 10.3390/jcm14155535

**Published:** 2025-08-06

**Authors:** Géraldine Désirée Sturz-Jantsch, Melanie Winter, Stefan Hajdu, Thomas Haider

**Affiliations:** Department of Orthopedics and Trauma Surgery, Medical University of Vienna, 1090 Wien, Austria

**Keywords:** calcaneal fracture, multiple-injured patients, concomitant spine injury, Böhler angle

## Abstract

**Background/Objectives**: The calcaneus is the most commonly injured tarsal bone, potentially resulting in long-term functional deficiencies and disability. The type of treatment mainly depends on fracture type and morphology. Treatment of these fractures can be challenging due to a limited soft tissue envelope and is frequently associated with complications. The aim of this study was to classify fracture types and identify factors associated with in-hospital complications. **Methods**: Patients with calcaneal fractures treated at our level I trauma center between 1997 and 2017 were included. Demographic data, comorbidities, fracture characteristics, type of treatment, complications and revisions, compliance and accompanying injuries were evaluated. **Results**: A total of 238 patients (m = 163, f = 75) at a mean age of 40 years sustaining either uni- or bilateral calcaneal fracture resulting in a total of 288 calcaneal fractures. Concomitant injuries were present in 103 patients (35.9%). Traumatic spine lesions were present in 21.9%. Complications were recorded in 59 fractures (20.5%). Open fractures were more likely to develop complications (76.0% vs. 15.2%, *p* < 0.001). Significant complication (33% vs. 14%, *p* < 0.001) and wound complication rates (29% vs. 10%, *p* < 0.001) were found in multiple-injured patients. All open fractures were surgically treated on the day of admission. In calcaneal fractures with a Böhler angle below 0 degrees, more complications were seen (33% vs. 17%, *p* < 0.05). **Conclusions**: High complication rates following calcaneal fractures were detected, with an increased likelihood in open fractures and in patients with multiple injuries. A negative Böhler angle was associated with worse outcomes.

## 1. Introduction

Calcaneal fractures account for 2% of all fractures among adults, and yet they are the most frequent fractures of tarsal bones [1]. Common causes are high-energy traumas, such as falls from height, suicidal or accidental events, or motor-vehicle accidents [2,3]. 

These injuries often involve the subtalar joint and approximately 60 to 80% present with intra-articular components [3]. In 15%, patients sustain open fractures, particularly in cases of high-energy trauma, and in 5–10% both calcanei are affected. Most calcaneal fractures are prevalent in male patients with an average age of 30 to 50 years [4].

In patients with high-energy trauma, calcaneal fractures are often accompanied by spine injuries [5,6]. Furthermore, calcaneal fractures often occur as concomitant injuries in patients after high-energy trauma with femur or pelvic fractures, long bone fractures of the lower extremity or injuries to the abdomen or thorax [7].

Due to its complex anatomy—being the largest tarsal bone and having an articular component—the majority of the axial load of the body weight affects the calcaneus. Therefore, injuries lead to changes in the mechanics and function of the foot, which can lead to impairment.

The optimal management and treatment of calcaneal fractures remain controversially discussed. There is an ongoing debate as to whether operative or nonoperative treatment strategies are to be preferred, particularly in non-displaced fractures. While surgical intervention may restore anatomy and joint congruity, it is associated with risk of complications, particularly soft tissue problems, infections and wound dehiscence [7,8,9]. The reported complication rates after open reduction and internal fixation (ORIF) vary widely in the literature, ranging from 10 to 40% depending on the surgical technique, patient characteristics and injury severity [7,10].

Several studies have attempted to identify factors associated with poor outcomes in patients with calcaneal fractures. These include open fractures, delayed surgical treatment, significant comorbidities and radiographic parameters, such as a severely reduced Böhler angle [6,11,12]. Patients who sustain calcaneal fractures due to suicide attempts or high-impact falls may present with psychiatric disorders, which are also linked to impaired compliance potentially associated with an increased risk for complications [13,14,15].

The aim of this study was to analyze a 20-year cohort of patients treated for calcaneal fractures at a level I trauma center and identify factors associated with in-hospital complications, particularly fracture characteristics, comorbidities, injury mechanisms and Böhler angle.

## 2. Materials and Methods

A retrospective chart review was performed including 238 patients treated for calcaneal fractures between 1997 and 2017 at a level I trauma center. All patients older than 14 years with radiologically confirmed calcaneal fractures were included. Patients secondarily transferred from other hospitals, with pathological fractures and with incomplete records were excluded. The local institutional review board approved the study protocol. Collected data included age, sex, injury mechanism (e.g., fall height > 3 m was defined as fall from great height), fracture characteristics (classified according to Zwipp et al. [16] and Sanders et al. [17]), open or closed status, comorbidities (e.g., diabetes, psychiatric conditions, substance use) and treatment type. Radiographs were reviewed to assess fracture type and measure the Böhler angle [18], defined as the tuber joint angle between a line from the anterior process of the calcaneus to the posterior articular facet on a lateral radiograph of the foot and a line from the posterior articular facet to the calcaneal tuberosity.

The primary outcome was the occurrence of in-hospital complications related to the fracture or its treatment, including infections, wound healing problems, hardware failure, thrombosis, pneumonia or compartment syndrome. Operative treatment, when performed, followed standard protocols, with open reduction and internal fixation via an extended lateral approach or external fixation. The timing of surgery (within vs. after 4 days of admission) was recorded.

The chi-square test was performed to compare continuous variables; an odds ratio (OR) with 95% confidence interval (CI) was calculated where appropriate. Statistical significance was set at *p* < 0.05. Data was analyzed using SPSS^®^ 27 (IBM Corp., Chicago, IL, USA). Continuous data is given as mean ± standard deviation if not stated otherwise.

## 3. Results

### 3.1. Patient Demographic

A total of 238 patients at a mean age of 40 (range 15 to 88) years presented with calcaneal fractures to our department between 1997 and 2017. The mean follow-up was 24 months, with a median of 12 months and a range from 0 months up to 12.5 years (SD 31 months). The majority of patients were male (n = 163, 68.5%). In 50 cases (17%) bilateral calcaneal fractures were diagnosed, accounting for a total of 288 calcaneal fractures. The most common trauma mechanism was a fall from a great height in 66.3% of cases (n = 191). This included a known suicide attempt in 34 patients (14.3%) (Table 1).

A comorbidity was documented in 47.9% of patients, including nicotine, alcohol and drug abuse, diabetes, cardiovascular diseases and/or a psychiatric history. Psychiatric disorders were documented in 11.3% of patients, including depression, schizophrenia and substance dependence. Additional injuries were present in 82 patients (35.9%). Traumatic spine lesions were present in 21.5% of fractures. In total, 25 fractures were classified as open fractures (8.7%), 205 were comminuted (71.2%) and 213 had an intra-articular component (74.0%). (Detailed fracture classification in Table 2).

We treated 37 fractures nonoperatively (12.9%). The remaining 251 fractures were subjected to surgical fixation, of which 92 fractures required open reduction (36.65%). Fracture fixation was primarily obtained with screws (55.0%), followed by plate osteosynthesis (23.5%) and external fixation (13.1%). Operative treatment followed standard protocols, with ORIF via extended lateral approach in closed fractures. In open fractures, initial debridement and irrigation were performed, followed by surgical fixation (either ORIF or external fixation).

The mean time to surgery was 5 days (range 0 to 33) and the mean surgery time was 1 h 34 min (SD ± 41 min). In closed fractures the mean time to surgery was 6 days (range 0 to 33). All open fractures were operated upon on the day of admission.

### 3.2. Complications

Complications were identified based on documentation in the patients’ medical records. They were recorded in 59 calcaneal fractures (20.5%). The most frequent complications were wound-related, occurring in 49 cases (17.0%) (Table 3).

Wound complications included superficial and deep wound infections, necrosis of the wound edges and delayed healing. Surgical revision was required in 24 of the 49 wound-related cases (49%). As expected, open fractures showed significantly more overall complications (76% vs. 15%, *p* < 0.001) and wound complication rates (72% vs. 12%, *p* < 0.001) compared to closed fractures. Open fractures were more likely to develop wound complications (OR 19.244, 95% CI [7.442–49.764]).

Overall, there was no difference in complication rates between nonoperative and operative treatment (18% vs. 21%, *p* = 0.735). This remained true when only closed fractures were considered (overall complications: 13% vs. 16%, *p* = 0.649; wound complications: 13% vs. 12%, *p* = 0.894).

Similarly, regarding technique for surgical fracture reduction, no significant difference in complication rates was observed between fractures treated with open versus closed reduction techniques (22% vs. 20%, *p* = 0.873). Psychiatric disorders were significantly associated with higher complication rates (84.2% vs. 17.0%, *p* < 0.001), while nicotine and diabetes did not show a statistically significant association. The increased complications in psychiatric patients may be multifactorial, including compliance, medication and trauma mechanism. While we did not observe a significant association between nicotine use or diabetes and complication rates, this may be due to the retrospective nature of the study and heterogeneous fracture severity. Previous studies have reported delayed wound healing and increased infection rates in patients with these comorbidities [8,9,12]. Although earlier studies have reported higher complication rates associated with operative treatment of calcaneal fractures—particularly due to wound healing problems following lateral approaches [7,8,9,10]—we did not find a statistically significant difference in complication rates between operative and nonoperative management. This may be explained by treatment selection bias: more-complex fractures were more likely to be treated surgically, while less severe cases were treated nonoperatively. This suggests that the underlying risk of complications may have already been higher in the operative group, which could explain the absence of treatment-specific differences. Moreover, the retrospective study design and lack of functional outcome data limit direct comparisons. While our results reflect in-hospital complications only, existing randomized controlled trials, such as the one by Griffin et al. [10], also found no clear advantage of surgical over nonoperative treatment in terms of long-term functional outcome. These findings support the notion that the benefit–risk ratio of surgical intervention should be carefully evaluated on a case-by-case basis.

Both age and bilateral fractures were not associated with higher complication frequencies.

Multiple-injured patients had a significantly higher rate of overall complication (33% vs. 14%, *p* < 0.001) and wound complications (29% vs. 10%, *p* < 0.001). Among open fractures, all patients received surgical treatment on the day of admission. When open fractures were excluded from the analysis, there was no significant difference in complication frequencies between patients treated within 4 days after admission and those treated after more than 4 days after admission (*p* = 0.112 for overall complications; *p* = 0.631 for wound complications).

**Figure 1:**
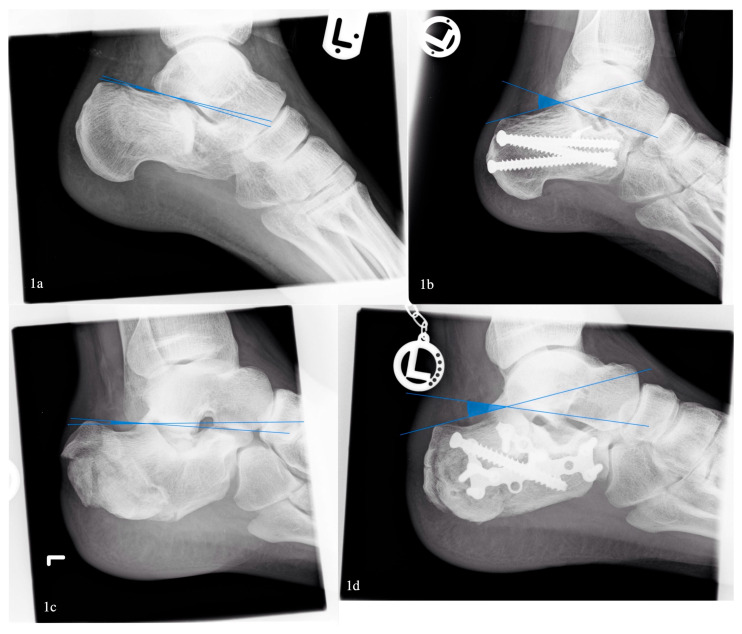
Comparison of pre- and postoperative Böhler angle. Pat. 1: (**1a**) 2° preoperative, (**1b**) 35° postoperative; Pat. 2: (**1c**) 5° preoperative, (**1d**) 23° postoperative.

A Böhler angle below 0 degrees was associated with higher overall complication rates (33% vs. 17%, *p* < 0.05), but no significant difference in wound complications was found (27% vs. 12%, *p* = 0.55) (Figure 1). After exclusion of open fractures, the association between a low Böhler angle and complications vanished.

### 3.3. Concomitant Spine Injury

Spinal injuries were documented in 21.5% of cases. In patients younger than 50 years old, calcaneal fractures were significantly more often associated with spine injuries (26% vs. 11%, *p* < 0.008). Also, bilateral calcaneal fractures were more often associated with spine injuries (58% vs. 27%, *p* < 0.001), as were open fractures (19% vs. 6%, *p* < 0.01). Patients with spine injuries had significantly higher rates of overall complications (31% vs. 17%, *p* < 0.05) and wound complications (27% vs. 13%, *p* < 0.05).

## 4. Discussion

Calcaneal fractures are often associated with poor outcomes and high complication rates. The aim of the present study was to provide a detailed demographic and clinical analysis of patients with calcaneal fractures and to identify factors associated with complications. Our main findings were that patients at our clinic sustaining calcaneal fractures were young and often presented with open and bilateral fractures with typical trauma mechanisms such as falls from a great height, often related to suicidal intent. With an average age of 40 years, our data correlate with findings in the literature [12]. As this represents the working-age population, calcaneal fractures in this age group can lead to prolonged occupational disability with a significant socioeconomic impact [2,19].

Furthermore, we detected a comparatively high prevalence of concomitant spine injuries in 21.5% of cases, which is higher than reported in most studies. Walters et al. [20] showed a prevalence of only 7%. A study by Wilson [14], with comparable patient demographics, stated an incidence of 22% of associated spine injuries, which is comparable to our results. In total, 87% of our patients with accompanying spine injuries were younger than 50 years. In a study by Boruah et al. [15], spinal fractures were found in 12% of patients sustaining calcaneal fractures. Furthermore, they observed that, due to the dispersion of forces, patients with concomitant calcaneal and spine injuries had lower rates of neurologic deficits. A study by Bohl et al. [11] from 2017 showed a rate of 23%. They explain their high number of spinal injuries through the fact that they routinely take spinal radiographs in patients with calcaneal fractures, following a recommendation from Browner et al. [21].

This may also explain the high correlation between calcaneal and spinal fractures observed in our data. Patients with high-impact trauma mechanisms, including falls from height or suicidal jumps, are typically admitted to our trauma emergency room. Due to the high-impact forces involved in their trauma mechanisms, these patients regularly undergo a full-body CT-scan. This may contribute to the increased detection of associated spine injuries. Moreover, bilateral and open fractures were significantly more commonly associated with spinal trauma in our cohort, underlining the importance of injury mechanism and force distribution. Our analysis also revealed a correlation between bilateral calcaneal fractures and spinal injuries (58%), which can also be explained by trauma mechanism. In accordance with Walsh et al. [13], psychiatric history was common in patients with calcaneal fractures. This may explain the high incidence of suicidal jumps as a trauma mechanism in patients with psychiatric history (83%) and a fall from a great height (14%). These patients also often sustain complications (43%). The high complication rate may be multifactorial, potentially influenced by reduced compliance. These findings highlight the clinical importance of early and comprehensive trauma assessment. Given the strong association between bilateral or open calcaneal fractures and spinal injuries, we recommend routine spinal imaging—particularly in younger patients and those with high-energy trauma mechanisms—regardless of their neurological symptoms. Early identification of spinal injuries is essential to avoid missed diagnoses, enable timely immobilization and optimize treatment planning [11,20]. In settings where full-body CT is not routinely performed, clinicians should be aware of the potential for associated spinal injuries in complex calcaneal fractures.

Our findings that a Böhler’s angle below 0 degrees is associated with a significantly higher overall complication rate are consistent with previous studies identifying a low Böhler’s angle as a predictor of poorer outcomes and higher complication rates in calcaneal fractures [8,9,17]. A severely decreased or negative Böhler’s angle reflects substantial collapse of the posterior facet and disruption of the calcaneal structure, which has been associated with greater soft tissue trauma and mechanical instability [8]. However, after excluding open fractures in our study, the association between Böhler’s angle and overall complications was no longer observed, suggesting that soft tissue injury severity, rather than bony deformity alone, may be the principal driver of complications. This aligns with recent studies emphasizing the critical role of soft tissue condition, particularly in high-energy injuries, on postoperative outcomes. This finding reinforces recent evidence that early assessment of soft tissue status should guide both surgical timing and treatment strategy [22].

Calcaneal fractures are known to result in hindfoot pain and reduced function due to cavovarus deformity [23]. Hindfoot deformities include shortening, widening and varus angulation, leading to altered gait patterns, subtalar arthrosis and impingement of either peroneal tendons or fibulocalcaneal structures [24]. In women especially, a widened heel can cause problems and result in shoe conflicts [13]. Another study, conducted by Griffin et al. [10], evaluated patient-reported outcomes two years after either nonoperative or operative treatment for calcaneal fractures. They found no significant differences in range of motion and heel width between the two groups. Nerve pain is also commonly reported after surgical treatment of calcaneal fractures, especially when using a lateral approach [25].

In our study, we did not find significant differences in comorbidities and complications, although these have been well documented in other studies [19]. This might be due to the heterogenous groups in terms of fracture characteristics and trauma mechanism. Patients sustaining a calcaneal fracture and presenting with a psychiatric disorder are probably more likely to have higher numbers of complications due to compliance and also due to trauma mechanism [13].

A strength of this study is its analysis of calcaneal fractures in a distinct trauma population with a high prevalence of bilateral and open calcaneal fractures, providing insight into injury patterns and associated spinal trauma. The observed associations between psychiatric comorbidities and trauma mechanisms provide relevant clinical context.

Limitations include the retrospective design, absence of functional outcome data and loss to follow-up, as the data collection relied on documentation performed during hospital stays.

## 5. Conclusions

This study demonstrates high complication rates in patients with calcaneal fractures. The most important factors associated with complications were open fractures, the presence of multiple injuries and a negative Böhler angle, as well as pre-existing psychiatric disorders. Surgical treatment was not associated with an increased complication likelihood when compared to nonoperative treatment.

## Figures and Tables

**Table 1:** Baseline characteristics.

	Patients
Demographic	
Number	238 (100)
Male	163 (68.4)
Female	75 (31.5)
Age at time of trauma (years)	40 (15–88)
Trauma Mechanism	
Fall > 3 m	191 (66.3)
MVA	37 (12.8)
Fall	36 (12.5)
Fall over stairs	12 (4.2)
Unknown	12 (4.2)

Values are presented as n, n (%) and mean (interquartile range). MVA: motor vehicle accident.

**Table 2:** Fracture characteristics.

	Patients
Fracture Type	
Closed	263 (91.3)
Open	25 (8.7)
Comminuted Fracture	
No	41 (14.2)
Yes	205 (71.2)
Unknown	42 (14.6)
Intra-Articular Fracture	
No	24 (8.3)
Yes	213 (74.0)
Unknown	51 (17.7)
Zwipp Classification	
2	11 (8.7)
3	5 (3.9)
4	4 (3.1)
5	5 (3.9)
6	2 (1.6)
7	10 (7.9)
8	15 (11.8)
9	8 (6.3)
10	16 (12.6)
11	45 (35.4)
12	6 (4.7)
Sanders Classification	
Type I	13 (4.5)
Type II	24 (8.3)
Type III	19 (6.6)
Type IV	64 (22.2)
Unknown	168 (58.3)
Böhler Angle	
Preoperative	14.7° (−50°–65°)
Postoperative	25.7° (−50°–55°)

Values are presented as n, n (%) and mean (interquartile range).

**Table 3:** Complications.

	Patients
Wound complications	49 (17.0)
Arthrodesis	6 (2.1)
Material failure	3 (1.0)
Hypoesthesia	1 (0.3)
No complication	229 (79.5)

Values are presented as n and n (%).

## Data Availability

The raw data supporting the conclusions of this article will be made available by the authors on request.

## Abbreviations

The following abbreviations are used in this manuscript:

CI	Confidence interval
OR	Odds ratio
ORIF	Open reduction and internal fixation
SD	Standard deviation
MVA	Motor vehicle accident

## References

[1] Molloy A.P., Lipscombe S.J. (2011). Hindfoot Arthrodesis for Management of Bone Loss Following Calcaneus Fractures and Nonunions. Foot Ankle Clin..

[2] Bruce J., Sutherland A. (2013). Surgical versus Conservative Interventions for Displaced Intra-Articular Calcaneal Fractures. Cochrane Database Syst. Rev..

[3] Palmersheim K., Hines B., Olsen B.L. (2012). Calcaneal Fractures: Update on Current Treatments. Clin. Podiatr. Med. Surg..

[4] Rixen D., Halfmann B., Fritzemeier C.-R. (2014). Fersenbeinfrakturen. Trauma. Berufskrankh..

[5] Worsham J.R., Elliott M.R., Harris A.M. (2016). Open Calcaneus Fractures and Associated Injuries. J. Foot Ankle Surg..

[6] Sanders R. (2000). Displaced Intra-Articular Fractures of the Calcaneus. J. Bone Jt. Surg. Am. Vol..

[7] Harvey E.J., Grujic L., Early J.S., Benirschke S.K., Sangeorzan B.J. (2001). Morbidity Associated with ORIF of Intra-Articular Calcaneus Fractures Using a Lateral Approach. Foot Ankle Int..

[8] Abidi N.A., Dhawan S., Gruen G.D.S., Vogt M.T., Conti S.F. (1998). Wound-Healing Risk Factors after Open Reduction and Internal Fixation of Calcaneal Fractures. Foot Ankle Int..

[9] Thordarson D.B., Krieger L.E. (1996). Operative vs. Nonoperative Treatment of Intra-Articular Fractures of the Calcaneus: A Prospective Randomized Trial. Foot Ankle Int..

[10] Griffin D., Parsons N., Shaw E., Kulikov Y., Hutchinson C., Thorogood M., Lamb S.E. (2014). Operative versus Non-Operative Treatment for Closed, Displaced, Intra-Articular Fractures of the Calcaneus: Randomised Controlled Trial. BMJ.

[11] Bohl D.D., Ondeck N.T., Samuel A.M., Diaz-Collado P.J., Nelson S.J., Basques B.A., Leslie M.P., Grauer J.N. (2017). Demographics, Mechanisms of Injury, and Concurrent Injuries Associated With Calcaneus Fractures: A Study of 14 516 Patients in the American College of Surgeons National Trauma Data Bank. Foot Ankle Spec..

[12] Bläsius F.M., Stockem L.E., Knobe M., Andruszkow H., Hildebrand F., Lichte P. (2022). Predictors for Wound Healing Complications and Prolonged Hospital Stay in Patients with Isolated Calcaneal Fractures. Eur. J. Trauma. Emerg. Surg..

[13] Walsh T.P., Vasudeva V., Sampang K., Platt S.R. (2021). Psychological Dysfunction Associated with Calcaneal Fractures. Injury.

[14] Wilson D.W. (1966). Functional Capacity Following Fractures of the Os Calcis. Can. Med. Assoc. J..

[15] Boruah T., Sareen A., Sreenivasan R., Kumar S., Chandra V., Patralekh M.K., Kumar R. (2022). Concomitant Spine and Calcaneum Fractures: A Possible Indication of Less Extensive Injury. Spinal Cord. Ser. Cases.

[16] Zwipp H., Tscherne H., Wülker N., Grote R. (1989). Intra-Articular Fracture of the Calcaneus. Classification, Assessment and Surgical Procedures. Unfallchirurg.

[17] Sanders R., Fortin P., DiPasquale T., Walling A. (1993). Operative Treatment in 120 Displaced Intraarticular Calcaneal Fractures. Results Using a Prognostic Computed Tomography Scan Classification. Clin. Orthop. Relat. Res..

[18] Lau B.C., Allahabadi S., Palanca A., Oji D.E. (2022). Understanding Radiographic Measurements Used in Foot and Ankle Surgery. J. Am. Acad. Orthop. Surg..

[19] Rammelt S., Zwipp H. (2014). Fractures of the Calcaneus: Current Treatment Strategies. Acta Chir. Orthop. Traumatol. Cech..

[20] Walters J.L., Gangopadhyay P., Malay D.S. (2014). Association of Calcaneal and Spinal Fractures. J. Foot Ankle Surg..

[21] Browner B.D., Jupiter J.B., Krettek C., Anderson P. (2009). Skeletal Trauma: Basic Science, Management, and Reconstruction.

[22] Schepers T., van Lieshout E.M.M., van Ginhoven T.M., Heetveld M.J., Patka P. (2008). Current Concepts in the Treatment of Intra-Articular Calcaneal Fractures: Results of a Nationwide Survey. Int. Orthop..

[23] Seaman T.J., Ball T.A. (2024). Pes Cavus. StatPearls [Internet].

[24] Su Y., Chen W., Zhang Q., Liu S., Zhang T., Zhang Y. (2014). Bony Destructive Injuries of the Calcaneus: Long-Term Results of a Minimally Invasive Procedure Followed by Early Functional Exercise: A Retrospective Study. BMC Surg..

[25] Haugsdal J., Dawson J., Phisitkul P. (2013). Nerve Injury and Pain after Operative Repair of Calcaneal Fractures: A Literature Review. Iowa Orthop. J..

